# The goat pan-genome reveals patterns of gene loss during domestication

**DOI:** 10.1186/s40104-024-01092-7

**Published:** 2024-10-05

**Authors:** Jiaxin Liu, Yilong Shi, Dongxin Mo, Lingyun Luo, Songsong Xu, Fenghua Lv

**Affiliations:** Frontiers Science Center for Molecular Design Breeding (MOE); State Key Laboratory of Animal Biotech Breeding; College of Animal Science and Technology, China Agricultural University, Beijing, 100193 China

**Keywords:** Domestication, Goat, Improvement, Pan-genome, Presence/absence variation

## Abstract

**Background:**

Unveiling genetic diversity features and understanding the genetic mechanisms of diverse goat phenotypes are pivotal in facilitating the preservation and utilization of these genetic resources. However, the total genetic diversity within a species can’t be captured by the reference genome of a single individual. The pan-genome is a collection of all the DNA sequences that occur in a species, and it is expected to capture the total genomic diversity of the specific species.

**Results:**

We constructed a goat pan-genome using map-to-pan assemble based on 813 individuals, including 723 domestic goats and 90 samples from their wild relatives, which presented a broad regional and global representation. In total, 146 Mb sequences and 974 genes were identified as absent from the reference genome (ARS1.2; GCF_001704415.2). We identified 3,190 novel single nucleotide polymorphisms (SNPs) using the pan-genome analysis. These novel SNPs could properly reveal the population structure of domestic goats and their wild relatives. Presence/absence variation (PAV) analysis revealed gene loss and intense negative selection during domestication and improvement.

**Conclusions:**

Our research highlights the importance of the goat pan-genome in capturing the missing genetic variations. It reveals the changes in genomic architecture during goat domestication and improvement, such as gene loss. This improves our understanding of the evolutionary and breeding history of goats.

**Supplementary Information:**

The online version contains supplementary material available at 10.1186/s40104-024-01092-7.

## Background

Under long-term adaptation to diverse environments and artificial selection, goats have evolved various phenotypes and specific genomic patterns compared with their wild ancestors [1]. In addition, domestic goats have incorporated genetic elements from multiple wild species, such as Caucasian tur (*Capra caucasica*) and Bezoar (*Capra aegagrus*) during the domestication and dispersal process [2]. Thus, the genomes of goats exhibit complex genetic characteristics [3]. Unveiling genetic diversity features and understanding the genetic mechanisms of diverse phenotypes of goats are pivotal in facilitating the preservation and utilization of these genetic resources.

Over the last decade, reference genomes have provided a roadmap and a fundamental framework for discovering genomic features. By aligning short reads to a reference genome, the genomic variations, such as single nucleotide polymorphisms (SNPs) and small insertions and deletions (INDELs), were identified, which elucidated the genetic mechanisms of the diverse phenotypes in humans [4], animals [5], and plants [6]. The current goat reference genome was generated from one individual and used in most goat genomic studies [7, 8]. However, the genetic diversity and genomic patterns within a species can’t be fully captured by the reference genome from a single individual [9]. The pan-genome is the nonredundant set of all DNA sequences in a specific species [10]. Pan-genome research in humans [10], animals [11], and plants [12] has provided novel scientific insights into the genetic diversity features and genetic mechanisms of diverse phenotypes. Compared with a reference genome from a single individual, a pan-genome could be applied to detect structural variations (SVs), such as copy number variations (CNVs) and presence/absence variations (PAVs) [13, 14]. These variations may significantly impact phenotype more than SNPs by altering gene dosage, interrupting coding sequence (CDS), and affecting long-range gene regulation [15–17]. The first goat pan-genome was assembled by Li et al. [18] using 10 individuals, including 5 samples from *Capra* genus and 5 individuals from sibling genus. However, the limited sample size can’t fully represent goats’ genetic and phenotypic diversity and constrain the detection of SVs, such as PAVs.

To obtain the comprehensive genome sequence and improve our understanding of the genomic characteristics of goats, the map-to-pan strategy was used to construct a goat pan-genome based on 723 domestic goats and 90 samples from their wild relatives, covering genetically, phylogenetically and geographically diverse samples (Additional file 1: Table S1). To identify the previously unknown SNPs, we aligned the reads that couldn’t be mapped to ARS1.2 to the nonreference sequences. PAVs were detected among the wild, native, and improved populations to reveal genetic changes through domestication and breeding history. This research will enhance our understanding of the changes in the genetic diversity and genomic architecture during domestication and improvement in goats.

## Methods

### Genome data collection, processing, and pan-genome construction

**Fig. 1:**
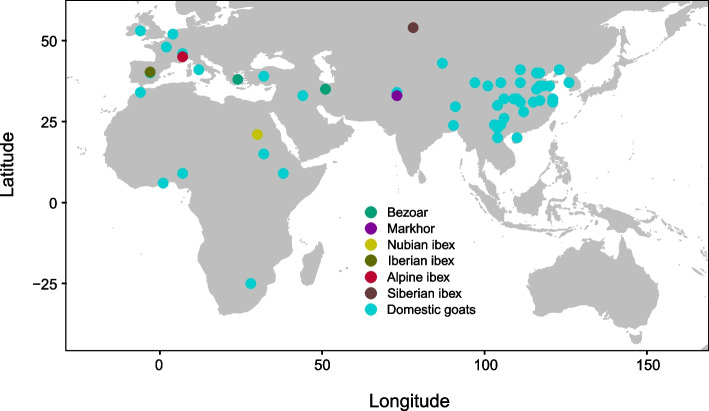
Geographical distribution of samples used for pan-genome construction

A total of 723 whole genome sequencing data of domestic goats from 85 populations and 90 samples from 6 wild goat species of *Capra* genus were downloaded from the National Center for Biotechnology Information Sequence Read Archive database (NCBI, https://www.ncbi.nlm.nih.gov) (Fig. 1, Table 1, Additional file 1: Table S1). The map-to-pan strategy was used to construct a goat pan-genome [19]. More specifically, raw Illumina reads were filtered to remove adapters and low-quality sequences using Trimmomatic (v0.39) [20] with parameters “SLIDINGWINDOW:4:15 MINLEN:50”. The high-quality reads of each individual were mapped to ARS1.2 using BWA (v0.7.12) [21] with the default parameters. The reads unmapped to ARS1.2 were extracted using SAMtools (v1.9) [22] with parameters "-b -f 4", "-b -f 68 -F 8" and "-b -f 132 -F 8", respectively. Subsequently, the unmapped reads were merged into a unified BAM file by SAMtools (v1.9) [22], followed by conversion of the BAM file to FASTQ format using bamtools (v2.5.2) (https://github.com/pezmaster31/bamtools). The FASTQ file was divided into paired FASTQ files corresponding to the forward and reverse strands utilizing an in-house Python script. Then, the paired unmapped reads were assembled using MaSuRCA (v4.1.0) [23] with parameters “USE_LINKING_MATES = 1, JF_SIZE = 5,000,000,000, FLYE_ASSEMBLY = 0”. Contigs assembled with a length exceeding 500 bp were retained for further analysis. We polished the contigs following 4 steps. Firstly, the Mummer (v4.0.0-beta2) [24] was employed to filter the initial contigs by aligning them to the ARS1.2. Contigs exhibiting an identity of ≥ 90% and a coverage of ≥ 80% were subsequently excluded. Secondly, the CD-HIT (v4.8.1) [25] was applied for filtering, where the assembly contigs were aligned against themselves, and contigs displaying an identity ≥ 90% and coverage ≥ 90% were removed. Thirdly, all contigs were carried out a search within the NCBI-NT database using BLASTN (v 2.14.0) [26] to discern sequences associated with archaea, viruses, bacteria, fungi, and viridiplantae, and identified sequences from these categories were eliminated. Finally, the Kraken2 (v2.0.9-beta) [27] was employed to classify and filter the remaining contigs using the kraken2-microbial database, encompassing sequences of archaea, bacteria, fungi, protozoa, viruses, and humans. The unclassified contigs were retained and integrated with ARS1.2 to assemble a goat pan-genome.

**Table 1:** Summary of sample in this study

Species	Category		Number of samples
Alpine ibex (*Capra ibex*)	Wild goats		34
Bezoar (*Capra aegagrus*)			28
Iberian ibex (*Capra pyrenaica*)			4
Markhor (*Capra falconeri*)			3
Nubian ibex (*Capra nubiana*)			3
Siberian ibex (*Capra sibirica*)			18
Goats (*Capra hircus*)	Native domestic goats		648
	Improved domestic goats	Dairy type	60
		Meat type	15

### Annotation of the nonreference sequences

The nonreference sequences were annotated using a pipeline that integrated three distinct methodologies: ab initio gene prediction, RNA-Seq, and protein homology [28]. Firstly, the repetitive sequences were identified by scanning a custom repeat library and Bovidae repeat library in RepeatMasker (v4.1.2-p1) [29] and masked in subsequent annotation analysis, in which a custom repeat library was built by screening the nonreference sequence using RepeatModeler (v2.0.3) [30]. Secondly, ab initio gene prediction was conducted using Augustus (v3.1.0) [31] under the Bovidae model with default parameters, and SNAP (2006-07-28) [32] with the guidance of MAKER2 (v2.31.8) [28]. Thirdly, we collected 37 RNA-Seq data from 28 tissues as transcript evidence (Additional file 1: Table S2). The RNA-Seq reads were filtered to remove the adapter and low-quality sequences using Trimmomatic (v0.39) [20]. The clean reads were aligned to the nonreference sequences using Hisat2 (v2.2.1) [33], and the gene models were subsequently constructed from the result of alignments using StringTie (v2.1.7) [34]. We further downloaded protein sequences of *Homo sapiens*, *Mus musculus*, *Canis lupus* *familiaris*, *Bos taurus*, *Sus scrofa*, *Ovis aries*, and *Capra hircus* from RefSeq, and the sequences were aligned to the nonreference sequences using Spaln (v3.0.3) [35]. Finally, we integrated gene predictions based on evidence of ab initio gene prediction, RNA-Seq, and protein homology following the MAKER2 pipeline [28], and a set of high-confidence gene models were obtained using MAKER2 (v2.31.8) [28]. We further investigated gene description by aligning the protein sequences to the UniProt database (https://ftp.uniprot.org/pub/databases) using BLASTP (v2.11.0) [26].

### Novel SNP calling and population genetic analysis

The reads of each sample that couldn't be mapped to ARS1.2 were mapped to the nonreference sequence using BWA-MEM (v0.7.17-r1188) [21] with the default parameters. The duplicated mapped reads were filtered using *MarkDuplicates* module in GATK (v4.1.9.0) [36]. Short variations (SNPs and INDELs) were identified by *HaplotypeCaller* module in GATK (v4.1.9.0) [36]. *GenomicsDBImport* module in GATK (v4.1.9.0) [36] was used to generate a database for GVCF files of all samples. And then, genotypes of variations were detected using the *GenotypeGVCFs* module in GATK (v4.1.9.0) [36]. SNPs were selected by the *SelectVariants* module in GATK (v4.1.9.0) [36] and filtered out potential false-positive loci using the *VariantFiltration* module of GATK (v4.1.9.0) [36] under the parameters “QD < 2.0 || MQ < 40.0 || FS > 60.0 || SOR > 3.0 || MQRankSum < −12.5 || ReadPosRankSum < −8.0”. Biallelic SNPs were extracted using VCFtools (v0.1.15) [37] with parameters “--minDP 2 --min-alleles 2 --max-alleles 2”. We further filtered out SNPs with the following criteria using PLINK (v1.90b6.26) [38]: (1) minor allele frequency (MAF) < 0.01; (2) SNP call rate < 0.5; (3) individual call rate < 0.5. After the quality control, 3,190 novel SNPs were retained for subsequent analysis.

To investigate features of these novel SNPs, we performed population genetic analysis using novel SNP datasets, such as principal component analysis (PCA), an approximately maximum likelihood phylogenetic tree, and model-based clustering. We performed the above analysis based on two datasets: one comprising 723 domestic goats and the other including all the 813 individuals (Additional file 1: Table S1). PCA was performed using PLINK (v1.90b6.26) [38]. An approximately maximum likelihood phylogenetic tree was constructed using the FastTree (v2.1.11) [39] with default parameters. The final tree topology was visualized using iTOL tool [40]. We performed model-based clustering to estimate the ancestry of each individual using the ADMIXTURE (v1.3.0) [41] with the number of ancestry kinships (*K*) set to 2–8.

### PAV calling and analysis

To investigate the characteristics of PAV in different populations, individuals with a minimum average sequencing depth of 10 × were retained for PAV calling [9, 13]. A total of 277 individuals were retained for PAV calling, including 85 wild, 121 native, and 71 improved goats (Additional file 1: Table S3). The presence or absence of each gene in each individual was determined using SGSGeneloss-based method [42]. We retrieved the longest transcript for each gene, considering it as the gene body, and subsequently calculated coverage based on this gene body [13]. If a minimum of two reads covered less than 5% of the exon regions, the gene was defined as absent in this individual; otherwise, it was classified as present [43].

We further investigated effects of artificial and natural selection, which were among the most important factors that reshaped the genome's architecture [44]. To identify genes under selection during domestication and improvement, we implemented PAV selection analysis between Bezoar (*n* = 24) and native goats (*n* = 121) to identify genes associated with domestication (Table 2). We also performed PAV selection analysis between native goats (*n* = 121) and improved dairy goats (*n* = 57) to identify genes related to improvement process (Table 2). Fisher’s exact test was conducted to determine the significance of the difference in the presence frequencies for each gene. A gene with a *P* value less than 0.005 was chosen as the putative gene.

**Table 2:** Candidate PAV genes associated with selection in goats

Category	Comparisons	Samples (Numbers)		Candidate genes
Domestication	Bezoar versus Native goats	Bezoar (24)		*CLEC2D* (*LOC102184901*), *FAM26F* (*LOC102181592*)
		Native goats (121)		
Improvement	Native goats versus Dairy goats	Native goats (121)		*GIMAP6* (*LOC108635866*), *CLECL1* (*LOC108636138*)
		Dairy goats (57)	Saanen goats (33)	
			Toggenburg goats (24)	

## Results

### Pan-genome of *Capra* genus

A total of 813 individuals were collected, including 723 domestic goats and 90 wild goats from Europe, Africa, and Asia (Fig. 1, Additional file 1: Table S1), and were used to assemble a goat pan-genome. The average sequence coverage of all the individuals was 16.40× (1.33×–48.64×). After removing contaminants and redundancies, 146 Mb sequences absent from ARS1.2 were retained. These nonreference sequences contained 133,959 contigs (501 bp ≤ length of contig ≤ 113,829 bp) with an average length of 1,091 bp. In addition, 974 protein-coding genes were predicted in the nonreference genome (Additional file 1: Table S4). Ultimately, the goat pan-genome, comprising the ARS1.2 and the nonreference sequences, harbored 25,743 genes (21,546 protein-coding genes and 4,197 other types of genes) covering DNA length up to 3,068,830,139 bp. We also compared the size and number of protein-coding genes of the goat pan-genome with other published goat reference genomes, such as CHIR_1.0 (*GCF_000317765.1*) and CVASU_BBG_1.0 (*GCA_004361675.1*). The genome size and number of protein-coding genes in the goat pan-genome were higher than the two reference genomes (Additional file 1: Table S7).

### Novel SNP calling and population genetic analysis

**Fig. 2:**
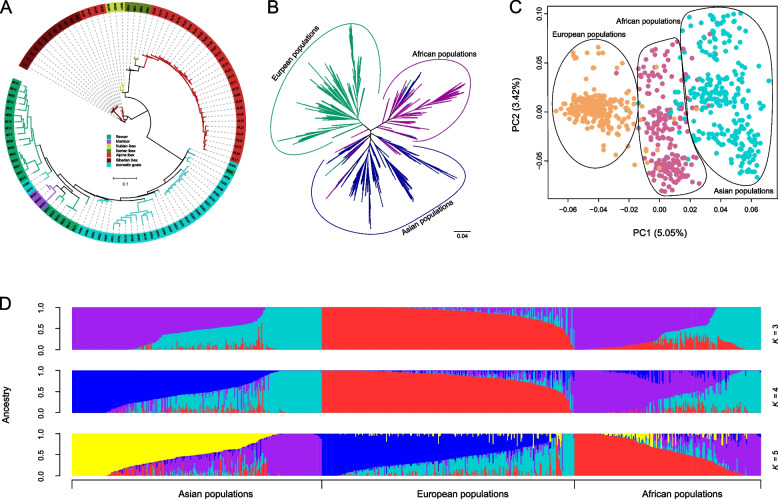
Population genetic analysis based on novel SNPs. **A** Maximum-likelihood tree based on novel SNPs for domestic goats and close wild relatives. **B** Maximum-likelihood tree based on novel SNPs for domestic goats. **C** Principal component analysis based on novel SNPs for domestic goats. **D** Model-based clustering of domestic goats with different numbers of ancestral kinships (*K* = 3, 4, and 5)

To identify missing SNPs in previous studies using a single reference genome, reads that couldn't be aligned to ARS1.2 were mapped to the nonreference sequences, and SNP calling was performed. We detected 3,190 high-quality novel SNPs in 813 samples. We conducted PCA, approximately-maximum-likelihood phylogenetic tree, and model-based clustering analysis using the novel SNP dataset. The results showed that samples from the same species tended to cluster (Fig. 2A, Additional files 2 and 3: Fig. S1 and S2). Most domestic goats were clustered based on their breeds (Additional file 4: Fig. S3), and domestic goats were split into 3 geographically structured groups (Asian, African, and European populations) (Fig. 2B–D, Additional files 2 and 3: Fig. S1 and S2). Population genetic analysis showed a close genetic relationship between Bezoar Ibex/Markhor and domestic goats (Fig. 2A, Additional files 2 and 3: Fig. S1 and S2). The pattern was consistent with previous studies using whole genome sequence datasets [1].

### Characterization of gene PAVs

**Fig. 3:**
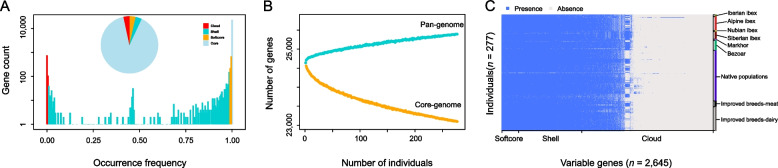
Characterization of gene PAVs and pan-genome model. **A** Pan-genome gene classification. **B** Pan-genome modeling. The upper and lower lines represent the pan and core-genome numbers, respectively. **C** The heatmap shows the PAVs of variable genes within wild, native, and improved dairy populations

A total of 277 individuals with an average sequence depth of more than 10× (Additional file 1: Table S3) were used to perform PAV calling. The presence frequency of each gene in all 277 individuals was calculated. In accordance with previous pan-genome studies [9, 13, 45], the genes were grouped into four categories (core, softcore, shell, cloud) according to their presence frequencies: core gene presenting in all individuals (100%); softcore gene presenting in more than 99% of individuals but less than 100% of individuals; shell gene presenting in 1%–99% individuals and cloud gene presenting in less than 1% individuals. Finally, 23,098 genes (89.73%) were found in all 277 individuals and were classified as core genes. The remaining 2,645 genes were classified as dispensable genes, including 914 softcore genes (3.55%), 867 shell genes (3.37%), and 864 cloud genes (3.36%) (Fig. 3A). The distribution pattern of gene PAVs was consistent with pan-genome studies of other species, such as chicken [13], *Brassica oleracea* [46], and arabidopsis [47]. It showed moderately high conserved genes (core and softcore genes) (Fig. 3A). The model of the pan-genome size by 200 iteratively randomly sampling individuals from 1 to 277 indicated an open pan-genome with an estimated total of 25,394 genes (Fig. 3B). The result suggested that the goat pan-genome assembled in this study didn’t reach saturation and could not include all or nearly all *Capra* genus gene contents. We also noted a different distribution of gene PAV across various species or populations (Fig. 3C). A striking feature was the decrease in gene presence from wild species to native populations and further from native populations to improved populations (Fig. 3C).

### Gene PAVs associated with domestication and improvement of goats

**Fig. 4:**
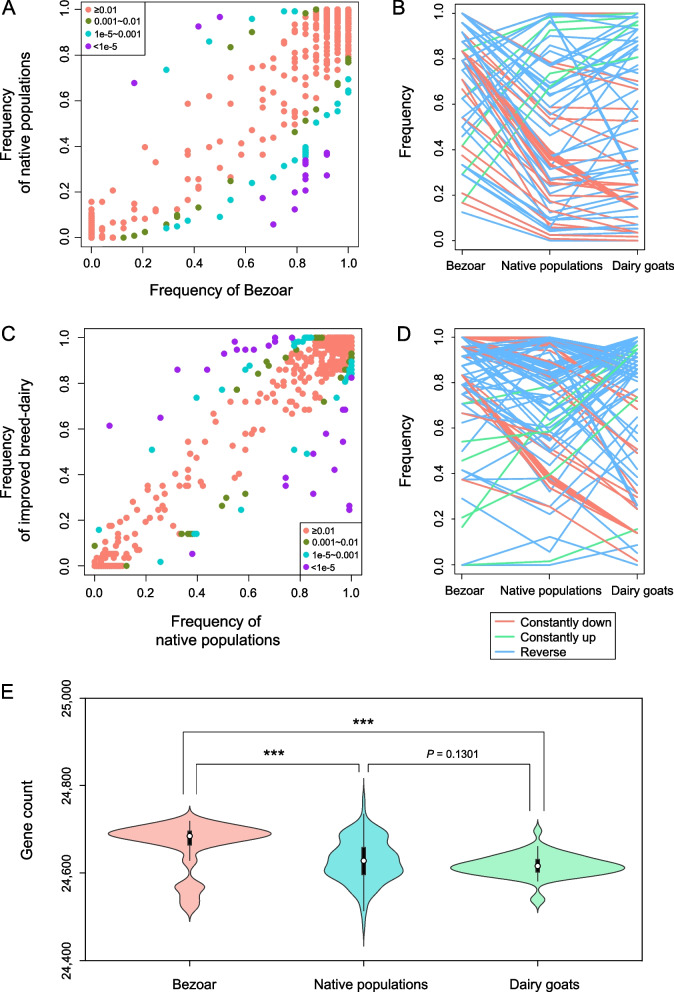
Gene PAVs associated with domestication and improvement of goats. **A** Scatter plots showing gene occurrence frequencies in wild (Bezoar) and native populations. **B** Occurrence frequency patterns of putative selected genes during domestication. **C** Scatter plots showing gene occurrence frequencies in native and improved populations. **D** Occurrence frequency patterns of putative selected genes during improvement. **E** Violin plots showing the number of detected genes in each individual within wild, native, and improved dairy populations. The significant difference of Mann–Whitney U test: ^***^P < 0.001

We compared gene presence between Bezoar and native goat populations (Table 2, Additional file 1: Table S2). The genes with higher frequencies in native goat populations than Bezoar population were considered as possible favorable genes, and genes with lower frequencies in native goat populations than Bezoar population were considered as possible unfavorable genes [9]. We identified 89 genes with significantly altered frequencies (Fig. 4A), comprising 15 favorable genes and 74 unfavorable genes (Fig. 4B). Annotation analysis indicated that all 89 genes were in ARS1.2 (Additional file 1: Table S5). Among the 15 favorable genes, *CLEC2D* (*LOC102184901*) was associated with immune and inflammatory responses (Table 2) and has been fixed in native goats (frequency = 99.17%) (Additional file 1: Table S5). We also detected genes related to immune and inflammatory responses in unfavorable genes, such as *FAM26F* (*LOC102181592*)*. FAM26F* that encoding tetraspanin-like membrane glycoprote [48] was detected in all Bezoar populations and 64.46% of native populations (Additional file 1: Table S5). We performed a comparative analysis of gene presence between native goats and dairy goats. Of note, 35 favorable genes (with a higher frequency in improved goat populations compared with native goat populations) and 72 unfavorable genes (with a lower frequency in improved goat populations compared with native goat populations) were identified (Fig. 4C and D, Additional file 1: Table S6). Genes associated with immune and inflammatory responses were also found in unfavorable genes, such as *GIMAP6* (*LOC108635866*) and *CLECL1* (*LOC108636138*). *GIMAP6* was detected in 96.69% of native goats and 54.39% of dairy goats (Additional file 1: Table S6) and has been reported to be associated with the regulation of autophagy, immune competence, and inflammation in mammals [49].

Notably, a prominent feature was the loss of genes during domestication and improvement (Fig. 4B and D). Specifically, 75 (83.15%) and 72 genes (67.29%) were identified as unfavorable genes, respectively (Additional file 1: Table S5 and S6). We further compared the gene count within each individual of the Bezoar population and the native goat and dairy goat populations. An average of 24,664, 24,623, and 24,615 genes were detected, showing a decreasing trend in gene number from wild species to native populations and from native populations to dairy populations (Fig. 4E).

## Discussion

This study used the map-to-pan strategy to construct a goat pan-genome. Taking into account the potential impact of sample size and representativeness on the pan-genome in humans and other organisms [13, 50, 51], we collected a diverse range of samples, covering almost all goats and their wild relatives across various geographic regions (Fig. 1, Additional file 1: Table S1), surpassing the scope of the previous study [18]. A total of 146 Mb nonreference sequences and 974 additional genes were identified as absent from ARS1.2. Notably, both indices were higher than those in the previous goat pan-genome study [18]. Utilizing the goat pan-genome, we identified 3,190 novel SNPs, indicating that the pan-genome can capture a greater diversity of genetic variations than a single reference genome.

Modeling of the goat pan-genome size revealed an open pan-genome (Fig. 3B). The result suggested that 277 samples in our PAV analysis were insufficient to capture the full spectrum of genetic diversity within goats. One potential reason was the limited sample size. Our study’s sample size was moderate compared with other animals, such as 268 individuals in chicken [13] and 250 individuals in pigs [11]. Nevertheless, the mosaic domestication of goats in the Fertile Crescent has led to genetically and geographically distinct goat populations since the Neolithic period, and continues to influence their diversity today [3, 52]. In addition, a high level of interspecies sequence variation has been observed compared with intraspecies in *Capra* genus [53], a high proportion of wild goats (31%, Additional file 1: Table S3) in our PAV analysis could contribute to the heterogeneity observed in genomic sequences. Therefore, by incorporating additional samples, particularly from improved and native breeds outside of China, PAV analysis will encompass a broader spectrum of goat gene contents.

Compared with other species, the goat pan-genome exhibited a higher proportion of core genes (89.73%), such as in pigs (75%) [54], chickens (76.32%) [13] and goose (75.86%) [55]. Although the proportion of core genes may decrease with an increase in sample size, 11% of variable genes within the goat pan-genome indicate substantial genomic potential for goat breeding. The PAV analysis unveiled gene loss events during domestication and improvement (Fig. 4). Comparable patterns have been observed in domesticated plants, such as tomato and cotton [9, 56]. The annotation of PAVs suggested that domestication and improvement may have influenced genomic features associated with immune and inflammatory responses [9, 56]. We also identified several immune and inflammation-related genes, including *CLEC2D*, *FAM26F*, *GIMAP6*, and *CLECL1* (Table 2), that may be related to the domestication and improvement of goats. *CLEC2D* was found to be fixed (frequency = 0.99) within native goat populations. It plays a role in regulating immune and inflammatory responses as a ligand of *CD161* receptor, and inhibiting the function of NK cells and T cells by its expression [57, 58]. *FAM26F* was observed to be fixed (frequency = 1.00) within Bezoar population. The gene could be activated by various infections, such as bacteria, viruses, and parasites, and expressed in various immune cells [48]. This observation is consistent with variable environments encountered by Bezoar. Bezoars are primarily distributed in a discontinuous range across Central Asia and the Caucasus, extending to southwestern Turkey [59]. Their natural habitats are characterized by harsh environmental conditions, including arid deserts and high-altitude rocky mountains [60]. *GIMAP6* and *CLECL1* exhibited a higher frequency in native goats than dairy goats (Additional file 1: Table S6) and are associated with immune responses [49, 61, 62]. This might also reflect the diverse environments encountered by native goats [53].

## Conclusion

We constructed a goat pan-genome based on 813 individuals, including 723 domestic goats and 90 samples from their wild relatives, which presented a broad genetic and geographical representativeness. A total of 146 Mb sequences and 974 protein-coding genes were identified as absent from ARS1.2. Additionally, 3,190 novel SNPs were identified from the nonreference sequences, which suggested that the goat pan-genome could capture more genetic variations. PAV analysis revealed evidence of gene loss during domestication and subsequent improvement processes. PAV selection analysis identified several genes related to immune regulation and inflammatory responses. This research enhances our understanding of the genomic changes throughout the history of goat breeding and highlight the importance of pan-genome in goat genomic studies.

## Supplementary Information

**Additional file 1: Table S1.** Summary of samples used in Pan-genome construction. **Table S2.** Summary of RNA samples for Pan-sequence Annotation. **Table S3.** Summary of samples used in PAV calling. **Table S4.** Protein-coding genes predicted in the non-reference genome of goat. **Table S5.** Gene list of significantly altered PAVs during goat domestication. **Table S6.** Gene list of significantly altered PAVs during goat improvement. **Table S7.** Comparison of the genome size and number of protein-coding genes between the goat pan-genome and other released genomes.

**Additional file 2: Fig.** **S1.** Principal component analysis based on novel SNPs for domestic goats and wild relatives.

**Additional file 3: Fig.** **S2.** Model-based clustering of domestic goats and close wild relatives with different numbers of ancestral kinships (*K* = 2, 3, 4, 5, 6, 7, and 8).

**Additional file 4: Fig.** **S3.** Maximum-likelihood tree based on novel SNPs for domestic goats and close wild relatives (clustered based on population, the node names consisted of the population names and the individual numbers, separated by the character "-".).

## Data Availability

The whole genome re-sequence data used for the study is publicly available under the sample accession numbers listed in Additional file 1: Table S1. All scripts used for this work were performed using open-source software tools and are available from the corresponding authors upon request.

## Abbreviations

CDS: Coding sequence
CNV: Copy number variation
INDELs: Small insertions and deletions
MAF: Minor allele frequency
PAV: Presence/absence variation
PCA: Principal component analysis
SNP: Single nucleotide polymorphism
SV: Structural variation

## References

[1] Denoyelle L, Talouarn E, Bardou P, Colli L, Alberti A, Danchin C, et al. VarGoats project: a dataset of 1159 whole-genome sequences to dissect *Capra hircus* global diversity. Genet Sel Evol. 2021;53:86. 34749642 10.1186/s12711-021-00659-6 PMC8573910

[2] Pogorevc N, Dotsev A, Upadhyay M, Sandoval-Castellanos E, Hannemann E, Simčič M, et al. Whole-genome SNP genotyping unveils ancestral and recent introgression in wild and domestic goats. Mol Ecol. 2024;33:e17190. 37909668 10.1111/mec.17190

[3] Daly KG, Delser PM, Mullin VE, Scheu A, Mattiangeli V, Teasdale MD, et al. Ancient goat genomes reveal mosaic domestication in the Fertile Crescent. Science. 2018;361:85–8. 29976826 10.1126/science.aas9411

[4] Fan S, Spence JP, Feng Y, Hansen MEB, Terhorst J, Beltrame MH, et al. Whole-genome sequencing reveals a complex African population demographic history and signatures of local adaptation. Cell. 2023;186:923–39. 36868214 10.1016/j.cell.2023.01.042 PMC10568978

[5] Li X, Yang J, Shen M, Xie X, Liu G, Xu Y, et al. Whole-genome resequencing of wild and domestic sheep identifies genes associated with morphological and agronomic traits. Nat Commun. 2020;11:2815. 32499537 10.1038/s41467-020-16485-1 PMC7272655

[6] Dang D, Guan Y, Zheng H, Zhang X, Zhang A, Wang H, et al. Genome wide association study and genomic prediction on plant architecture traits in sweet corn and waxy corn. Plants (Basel). 2023;12:303. 36679015 10.3390/plants12020303 PMC9867343

[7] Dong Y, Xie M, Jiang Y, Xiao N, Du X, Zhang W, et al. Sequencing and automated whole-genome optical mapping of the genome of a domestic goat (*Capra hircus*). Nat Biotechnol. 2013;31:135–41. 23263233 10.1038/nbt.2478

[8] Bickhart DM, Rosen BD, Koren S, Sayre BL, Hastie AR, Chan S, et al. Single-molecule sequencing and chromatin conformation capture enable de novo reference assembly of the domestic goat genome. Nat Genet. 2017;49:643–50. 28263316 10.1038/ng.3802 PMC5909822

[9] Gao L, Gonda I, Sun H, Ma Q, Bao K, Tieman DM, et al. The tomato pan-genome uncovers new genes and a rare allele regulating fruit flavor. Nat Genet. 2019;51:1044–51. 31086351 10.1038/s41588-019-0410-2

[10] Li Q, Tian S, Yan B, Liu C, Lam T-W, Li R, et al. Building a Chinese pan-genome of 486 individuals. Commun Bio. 2021;4:1016. 34462542 10.1038/s42003-021-02556-6 PMC8405635

[11] Li Z, Liu X, Wang C, Li Z, Jiang B, Zhang R, et al. The pig pangenome provides insights into the roles of coding structural variations in genetic diversity and adaptation. Genome Res. 2023;33:1833–47. 37914227 10.1101/gr.277638.122 PMC10691484

[12] Torkamaneh D, Lemay MA, Belzile F. The pan-genome of the cultivated soybean (PanSoy) reveals an extraordinarily conserved gene content. Plant Biotechnol J. 2021;19:1852–62. 33942475 10.1111/pbi.13600 PMC8428833

[13] Wang K, Hu H, Tian Y, Li J, Scheben A, Zhang C, et al. The chicken pan-genome reveals gene content variation and a promoter region deletion in *IGF2BP1* affecting body size. Mol Biol Evol. 2021;38:5066–81. 34329477 10.1093/molbev/msab231 PMC8557422

[14] Gong Y, Li Y, Liu X, Ma Y, Jiang L. A review of the pangenome: how it affects our understanding of genomic variation, selection and breeding in domestic animals? J Anim Sci Biotechnol. 2023;14:73. 10.1186/s40104-023-00860-1 PMC10161434 37143156

[15] Sudmant PH, Mallick S, Nelson BJ, Hormozdiari F, Krumm N, Huddleston J, et al. Global diversity, population stratification, and selection of human copy-number variation. Science. 2015;349:aab3761. 26249230 10.1126/science.aab3761 PMC4568308

[16] Alkan C, Coe BP, Eichler EE. Genome structural variation discovery and genotyping. Nat Rev Genet. 2011;12:363–76. 21358748 10.1038/nrg2958 PMC4108431

[17] Abel HJ, Larson DE, Regier AA, Chiang C, Das I, Kanchi KL, et al. Mapping and characterization of structural variation in 17,795 human genomes. Nature. 2020;583:83–9. 32460305 10.1038/s41586-020-2371-0 PMC7547914

[18] Li R, Fu W, Su R, Tian X, Du D, Zhao Y, et al. Towards the complete goat pan-genome by recovering missing genomic segments from the reference genome. Front Genet. 2019;10:1169. 31803240 10.3389/fgene.2019.01169 PMC6874019

[19] Wang W, Mauleon R, Hu Z, Chebotarov D, Tai S, Wu Z, et al. Genomic variation in 3,010 diverse accessions of Asian cultivated rice. Nature. 2018;557:43–9. 29695866 10.1038/s41586-018-0063-9 PMC6784863

[20] Bolger AM, Lohse M, Usadel B. Trimmomatic: a flexible trimmer for Illumina sequence data. Bioinformatics. 2014;30:2114–20. 24695404 10.1093/bioinformatics/btu170 PMC4103590

[21] Li H, Durbin R. Fast and accurate short read alignment with Burrows-Wheeler transform. Bioinformatics. 2009;25:1754–60. 19451168 10.1093/bioinformatics/btp324 PMC2705234

[22] Danecek P, Bonfield JK, Liddle J, Marshall J, Ohan V, Pollard MO, et al. Twelve years of SAMtools and BCFtools. GigaScience. 2021;10:giab008. 33590861 10.1093/gigascience/giab008 PMC7931819

[23] Sakaue S, Kanai M, Tanigawa Y, Karjalainen J, Kurki M, Koshiba S, et al. A cross-population atlas of genetic associations for 220 human phenotypes. Nat Genet. 2021;53:1415–24. 34594039 10.1038/s41588-021-00931-x PMC12208603

[24] Marçais G, Delcher AL, Phillippy AM, Coston R, Salzberg SL, Zimin A. MUMmer4: a fast and versatile genome alignment system. PLoS Comput Biol. 2018;14:e1005944. 29373581 10.1371/journal.pcbi.1005944 PMC5802927

[25] Fu L, Niu B, Zhu Z, Wu S, Li W. CD-HIT: accelerated for clustering the next-generation sequencing data. Bioinformatics. 2012;28:3150–2. 23060610 10.1093/bioinformatics/bts565 PMC3516142

[26] Camacho C, Coulouris G, Avagyan V, Ma N, Papadopoulos J, Bealer K, et al. BLAST+: architecture and applications. BMC Bioinformatics. 2009;10:421. 20003500 10.1186/1471-2105-10-421 PMC2803857

[27] Wood DE, Lu J, Langmead B. Improved metagenomic analysis with Kraken 2. Genome Biol. 2019;20:257. 31779668 10.1186/s13059-019-1891-0 PMC6883579

[28] Holt C, Yandell M. MAKER2: an annotation pipeline and genome-database management tool for second-generation genome projects. BMC Bioinformatics. 2011;12:491. 22192575 10.1186/1471-2105-12-491 PMC3280279

[29] Tarailo-Graovac M, Chen N. Using RepeatMasker to identify repetitive elements in genomic sequences. Curr Protoc Bioinformatics. 2009;25:4.10.1-14. 10.1002/0471250953.bi0410s25 19274634

[30] Flynn JM, Hubley R, Goubert C, Rosen J, Clark AG, Feschotte C, et al. RepeatModeler2 for automated genomic discovery of transposable element families. Proc Natl Acad Sci USA. 2020;117:9451–7. 32300014 10.1073/pnas.1921046117 PMC7196820

[31] Stanke M, Schöffmann O, Morgenstern B, Waack S. Gene prediction in eukaryotes with a generalized hidden Markov model that uses hints from external sources. BMC Bioinformatics. 2006;7:62. 16469098 10.1186/1471-2105-7-62 PMC1409804

[32] Korf I. Gene finding in novel genomes. BMC Bioinformatics. 2004;5:59. 15144565 10.1186/1471-2105-5-59 PMC421630

[33] Kim D, Langmead B, Salzberg SL. HISAT: a fast spliced aligner with low memory requirements. Nat Methods. 2015;12:357–60. 25751142 10.1038/nmeth.3317 PMC4655817

[34] Pertea M, Pertea GM, Antonescu CM, Chang T-C, Mendell JT, Salzberg SL. StringTie enables improved reconstruction of a transcriptome from RNA-seq reads. Nat Biotechnol. 2015;33:290–5. 25690850 10.1038/nbt.3122 PMC4643835

[35] Iwata H, Gotoh O. Benchmarking spliced alignment programs including Spaln2, an extended version of Spaln that incorporates additional species-specific features. Nucleic Acids Res. 2012;40:e161. 22848105 10.1093/nar/gks708 PMC3488211

[36] DePristo MA, Banks E, Poplin R, Garimella KV, Maguire JR, Hartl C, et al. A framework for variation discovery and genotyping using next-generation DNA sequencing data. Nat Genet. 2011;43:491–8. 21478889 10.1038/ng.806 PMC3083463

[37] Danecek P, Auton A, Abecasis G, Albers CA, Banks E, DePristo MA, et al. The variant call format and VCFtools. Bioinformatics. 2011;27:2156–8. 21653522 10.1093/bioinformatics/btr330 PMC3137218

[38] Purcell S, Neale B, Todd-Brown K, Thomas L, Ferreira MAR, Bender D, et al. PLINK: A tool set for whole-genome association and population-based linkage analyses. Am J Hum Genet. 2007;81:559–75. 17701901 10.1086/519795 PMC1950838

[39] Price MN, Dehal PS, Arkin AP. FastTree: computing large minimum evolution trees with profiles instead of a distance matrix. Mol Biol Evol. 2009;26:1641–50. 19377059 10.1093/molbev/msp077 PMC2693737

[40] Letunic I, Bork P. Interactive tree of life (iTOL) v3: an online tool for the display and annotation of phylogenetic and other trees. Nucleic Acids Res. 2016;44:W242–5. 27095192 10.1093/nar/gkw290 PMC4987883

[41] Alexander DH, Novembre J, Lange K. Fast model-based estimation of ancestry in unrelated individuals. Genome Res. 2009;19:1655–64. 19648217 10.1101/gr.094052.109 PMC2752134

[42] Golicz AA, Martinez PA, Zander M, Patel DA, Van De Wouw AP, Visendi P, et al. Gene loss in the fungal canola pathogen Leptosphaeria maculans. Funct Integr Genomics. 2015;15:189–96. 25421464 10.1007/s10142-014-0412-1

[43] Golicz AA, Bayer PE, Barker GC, Edger PP, Kim H, Martinez PA, et al. The pangenome of an agronomically important crop plant *Brassica oleracea*. Nat Commun. 2016;7:13390. 27834372 10.1038/ncomms13390 PMC5114598

[44] Saravanan KA, Panigrahi M, Kumar H, Bhushan B, Dutt T, Mishra BP. Selection signatures in livestock genome: a review of concepts, approaches and applications. Livest Sci. 2020;241:104257.

[45] Gordon SP, Contreras-Moreira B, Woods DP, Des Marais DL, Burgess D, Shu S, et al. Extensive gene content variation in the *Brachypodium distachyon* pan-genome correlates with population structure. Nat Commun. 2017;8:2184. 29259172 10.1038/s41467-017-02292-8 PMC5736591

[46] Li X, Wang Y, Cai C, Ji J, Han F, Zhang L, et al. Large-scale gene expression alterations introduced by structural variation drive morphotype diversification in *Brassica oleracea*. Nat Genet. 2024;56:517–29. 38351383 10.1038/s41588-024-01655-4 PMC10937405

[47] Kang M, Wu H, Liu H, Liu W, Zhu M, Han Y, et al. The pan-genome and local adaptation of *Arabidopsis thaliana*. Nat Commun. 2023;14:6259. 37802986 10.1038/s41467-023-42029-4 PMC10558531

[48] Malik U, Javed N. FAM26F: An enigmatic protein having a complex role in the immune system. Int Rev Immunol. 2023;42:247–57. 27645024 10.1080/08830185.2016.1206098

[49] Yao Y, Jiang P, Chao BN, Cagdas D, Kubo S, Balasubramaniyam A, et al. *GIMAP6* regulates autophagy, immune competence, and inflammation in mice and humans. J Exp Med. 2022;219:e20201405. 35551368 10.1084/jem.20201405 PMC9111091

[50] Li R, Li Y, Zheng H, Luo R, Zhu H, Li Q, et al. Building the sequence map of the human pan-genome. Nat Biotechnol. 2010;28:57–63. 19997067 10.1038/nbt.1596

[51] Sherman RM, Forman J, Antonescu V, Puiu D, Daya M, Rafaels N, et al. Assembly of a pan-genome from deep sequencing of 910 humans of African descent. Nat Genet. 2019;51:30–5. 30455414 10.1038/s41588-018-0273-y PMC6309586

[52] Wu M, Wang D, Li M, Lv F. Artificial selection shapes the lower genomic diversity and higher selective pressures on the sex chromosomes of domestic animals. Sci China Life Sci. 2024;67:1072–5. 38277069 10.1007/s11427-023-2478-5

[53] Zheng Z, Wang X, Li M, Li Y, Yang Z, Wang X, et al. The origin of domestication genes in goats. Sci Adv. 2020;6:eaaz5216. 32671210 10.1126/sciadv.aaz5216 PMC7314551

[54] Tian X, Li R, Fu W, Li Y, Wang X, Li M, et al. Building a sequence map of the pig pan-genome from multiple de novo assemblies and Hi-C data. Sci China Life Sci. 2020;63:750–63. 31290097 10.1007/s11427-019-9551-7

[55] Gao G, Zhang H, Ni J, Zhao X, Zhang K, Wang J, et al. Insights into genetic diversity and phenotypic variations in domestic geese through comprehensive population and pan-genome analysis. J Anim Sci Biotechnol. 2023;14:150. 38001525 10.1186/s40104-023-00944-y PMC10675864

[56] Li J, Yuan D, Wang P, Wang Q, Sun M, Liu Z, et al. Cotton pan-genome retrieves the lost sequences and genes during domestication and selection. Genome Biol. 2021;22:119. 33892774 10.1186/s13059-021-02351-w PMC8063427

[57] Lai JJ, Cruz FM, Rock KL. Immune sensing of cell death through recognition of histone sequences by C-Type Lectin-Receptor-2d causes inflammation and tissue injury. Immunity. 2020;52:123–35. 31859049 10.1016/j.immuni.2019.11.013 PMC6962543

[58] Mathewson ND, Ashenberg O, Tirosh I, Gritsch S, Perez EM, Marx S, et al. Inhibitory CD161 receptor identified in glioma-infiltrating T cells by single-cell analysis. Cell. 2021;184:1281–98. 33592174 10.1016/j.cell.2021.01.022 PMC7935772

[59] Amills M, Capote J, Tosser-Klopp G. Goat domestication and breeding: a jigsaw of historical, biological and molecular data with missing pieces. Anim Genet. 2017;48:631–44. 28872195 10.1111/age.12598

[60] Weinberg P, Ambarli H. *Capra aegagrus*. The IUCN Red List of Threatened Species. 2020;e.T3786A22145942. 10.2305/IUCN.UK.2020-2.RLTS.T3786A22145942.en.

[61] Pascall JC, Webb LMC, Eskelinen E-L, Innocentin S, Attaf-Bouabdallah N, Butcher GW. GIMAP6 is required for T cell maintenance and efficient autophagy in mice. PLoS ONE. 2018;13:e0196504. 29718959 10.1371/journal.pone.0196504 PMC5931655

[62] Ryan EJ, Marshall AJ, Magaletti D, Floyd H, Draves KE, Olson NE, et al. Dendritic cell-associated lectin-1: A novel dendritic cell-associated, C-type lectin-like molecule enhances T cell secretion of IL-41. J Immunol. 2002;169:5638–48. 12421943 10.4049/jimmunol.169.10.5638

