# Supplementary figures and images for: The goat pan-genome reveals patterns of gene loss during domestication
Source: J Anim Sci Biotechnol. 2024 Oct 5;15:132. doi: 10.1186/s40104-024-01092-7 (PMC11453020; doi:10.1186/s40104-024-01092-7)

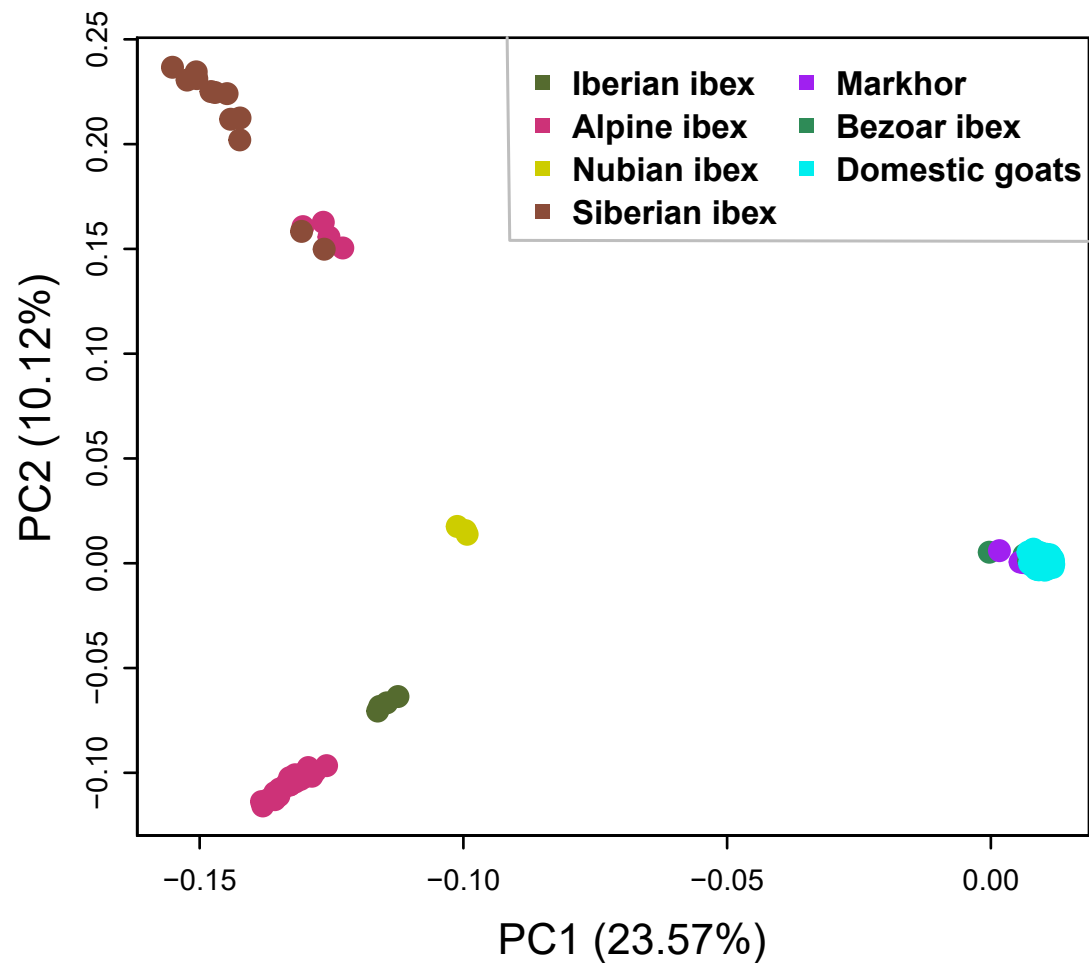

Supplement: Supplementary file 2 — Additional file 2: Fig. S1. Principal component analysis based on novel SNPs for domestic goats and wild relatives. [file 40104_2024_1092_MOESM2_ESM.pdf]

Ancestry

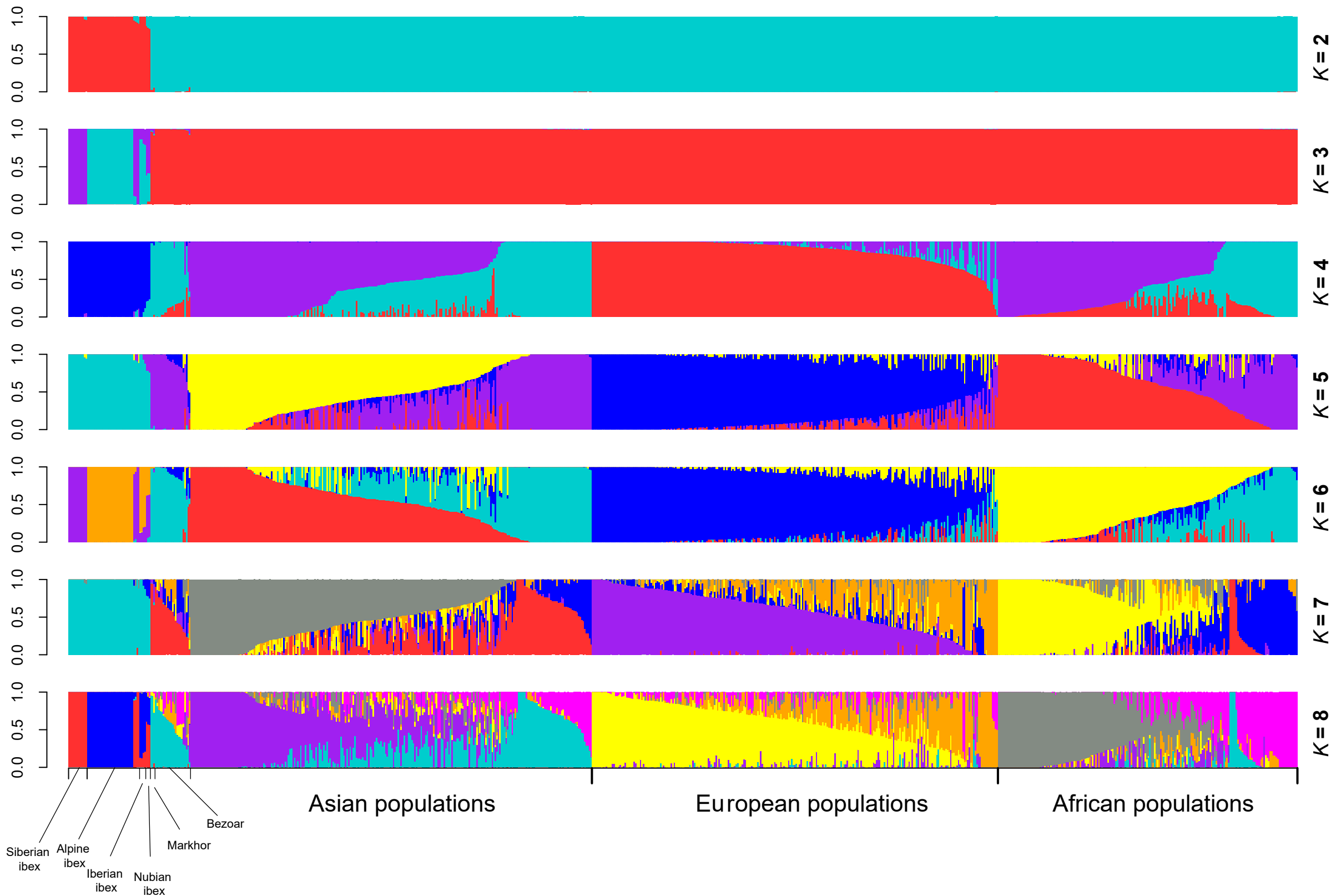

Supplement: Supplementary file 3 — Additional file 3: Fig. S2. Model-based clustering of domestic goats and close wild relatives with different numbers of ancestral kinships (K = 2, 3, 4, 5, 6, 7, and 8). [file 40104_2024_1092_MOESM3_ESM.pdf]

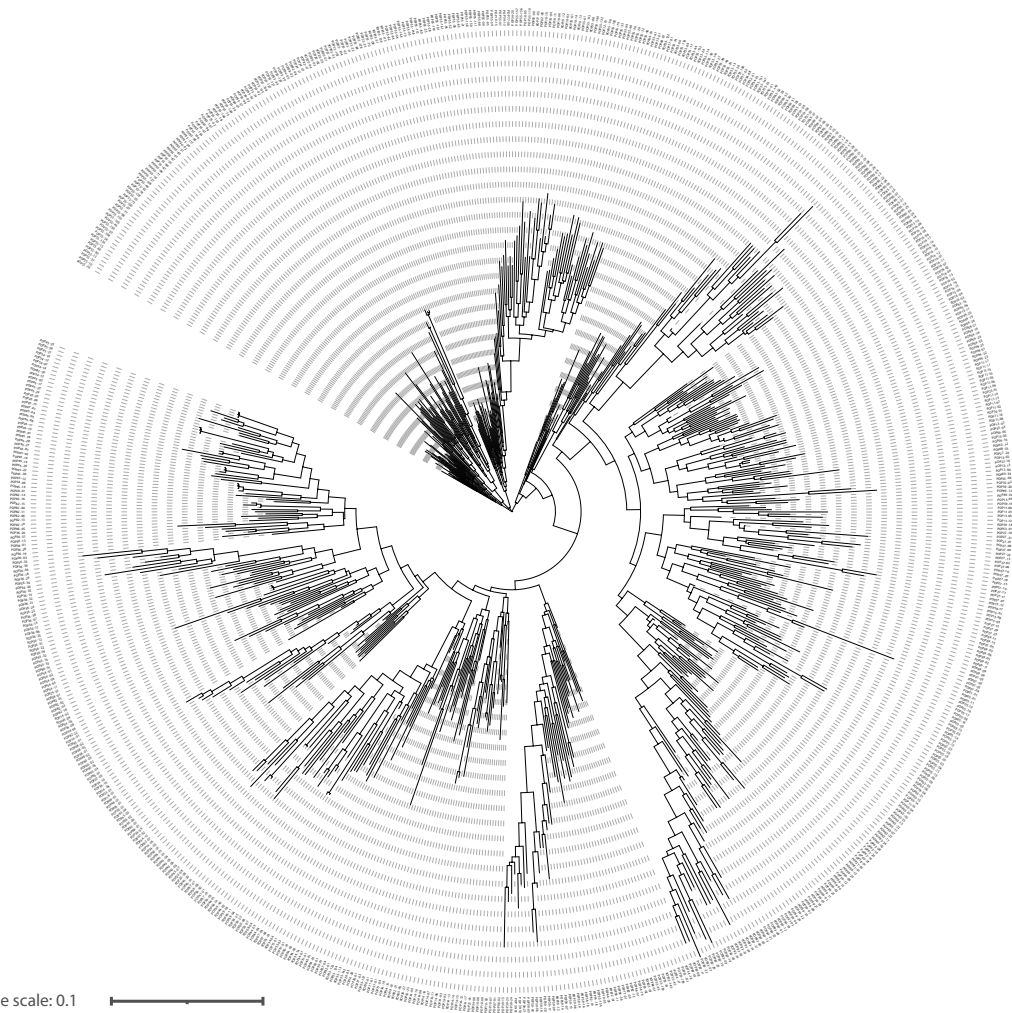

Tree scale: 0.1

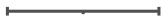

Supplement: Supplementary file 4 — Additional file 4: Fig. S3. Maximum-likelihood tree based on novel SNPs for domestic goats and close wild relatives (clustered based on population, the node names consisted of the population names and the individual numbers, separated by the character "-".). [file 40104_2024_1092_MOESM4_ESM.pdf]
